# *Bacillus cereus Sensu Lato* Genomes: Basis for Identifying Anthrax Disease Strain Sources

**DOI:** 10.1128/genomeA.00790-13

**Published:** 2013-10-03

**Authors:** Simon Silver

**Affiliations:** University of Illinois, Chicago, Illinois, USA

## TEXT

Why would one want to sequence the genomes of 94 additional, mostly soil and water isolates of *Bacillus cereus sensu lato* (1)? The answer is of course to be able to make a rapid analysis during a bioterrorism event, to develop a portable kit for the rapid identification of anthrax spores (by proteomic analysis), and to determine their precise origin (by means of DNA genomic polymorphisms). The draft genome sequences of 94 environmental *Bacillus cereus sensu lato* strains were deposited in GenBank (1) to facilitate the identification of the closely related subspecies *B. cereus*, *Bacillus anthracis, Bacillus mycoides*, and *Bacillus thuringiensis* and toxicity determinants. Strains of this species often harbor pathogenicity plasmids and show different envirotypes and pathotypes.

Some of these nonclinical environmental isolates harbor homologs to the *B. anthracis* anthrax pathogenicity plasmids pXO1 (192 kb) and pXO2 (96 kb) (2). The presence of these plasmids is noted by Van der Auwera et al. (1) but not listed in the corresponding GenBank deposits. The strains have neither, both, or one of the plasmids.

There were already numerous related genome deposits made in GenBank over the last 10 years (3, 4), both as draft genomes as those mentioned here and as complete genomes, some with published reports and comparative analyses (5–7). The authors promise subsequent in-depth analysis. The purpose is to understand the strain diversity of the bioterrorism pathogen *B. anthracis* and how to distinguish it from the common soil microbe *B. cereus* and the insecticidal microbe *B. thuringiensis*. A search of previous GenBank genome projects lists 7 complete genomes and 22 draft genomes available in contigs for *B. anthracis*, including several versions of the laboratory *B. anthracis* Ames strain. There are 11 complete genomes and 17 draft genomes deposited for *B. thuringiensis*; for *B. cereus*, 13 complete genomes are listed, including one labeled as “biovar *anthracis*,” and an additional 31 draft genomes before this added 94, for a total of 195 available genomes.

The recognized related *sensu lato* species are so similar that they could share a single species name based on sequence similarities and traditional microbiological phenotypes (5–7). However, different ecotypes and pathotypes that are important to humans call for keeping separate names, so “*sensu lato*” is phylogenetically meaningful and “*sensu stricto*” is how to tell them apart.

Table 1 in the related article by Van der Auwera et al. (1) lists the 44 strains with GenBank accession numbers listed in reference 2 as containing or not containing plasmid pXO1- or pXO2-like genes. The sources are soils or water samples, many from Europe. The other 50 strains, their origins, and plasmid contents are also listed.

The sequence data are now all available in GenBank, and there is no bioterrorism danger; this is purely defensive biological warfare research. Together with another report (8) of a matrix-assisted laser desorption ionization–time of flight mass spectrometry (MALDI-TOF MS) proteomic analysis of proteins directly from anthrax spores (which would allow for identification with 30-min analysis), the data here presumably provide background strain source identification for the small annual number of natural animal (some in Texas) and a few human anthrax disease cases and future bioterrorism threats. Rapid recognition allows for the successful decontamination of sources and antibiotic treatment of patients, as required by the military and police.
